# Serum Soluble Corin Deficiency Predicts Major Disability within 3 Months after Acute Stroke

**DOI:** 10.1371/journal.pone.0163731

**Published:** 2016-09-22

**Authors:** Weidong Hu, Shi Chen, Yulin Song, Fangfang Zhu, Jijun Shi, Xiujie Han, Dan Zhou, Zhongwen Zhi, Fuding Zhang, Yun Shen, Juanjuan Ma, Chun-Feng Liu, Hao Peng

**Affiliations:** 1 Department of Neurology, the Second Affiliated Hospital of Soochow University, Suzhou, China; 2 School of Nursing, Medical College of Soochow University, Suzhou, China; 3 Department of Neurology, Anshan Changda Hospital, Anshan, China; 4 Department of Emergency, the Affiliated Hospital of Xuzhou Medical University, Xuzhou, China; 5 Institute of Neuroscience, Soochow University, Suzhou, China; 6 Department of Epidemiology, School of Public Health, Medical College of Soochow University, Suzhou, China; Johns Hopkins University Bloomberg School of Public Health, UNITED STATES

## Abstract

**Objective:**

Serum soluble corin has been associated with stroke. However, whether it is associated with stroke prognosis has not yet been studied. Therefore, we aimed to study the association of serum soluble corin with risk of poor outcomes within 3 months after stroke.

**Methods:**

We followed 522 stroke patients for 3 months to identify major disability, death and vascular events. Serum soluble corin was measured at baseline for all participants. Logistic regression was used to examine the associations of baseline serum soluble corin with outcomes of stroke, adjusting for age, sex, baseline NIHSS score, hours from onset to hospitalization, smoking, drinking, hypertension, diabetes, coronary heart disease, atrial fibrillation, family history of stroke, and stroke subtype.

**Results:**

Patients with high corin had a significantly lower crude risk for the composite outcome of major disability or death (OR = 0.64, 95%CI: 0.43–0.96) than patients with low corin (the lowest tertile). After adjustment for age and baseline NIHSS score, patients with high corin still had a significantly lower risk for the composite outcome of major disability or death (OR = 0.60, 95%CI: 0.36–0.99). This association became bottom line significant after additionally adjusting for other conventional factors (OR = 0.61, *P* = 0.058). No association was found between serum soluble corin and other composite outcomes.

**Conclusion:**

Serum soluble corin deficiency predicted risk for major disability within 3 months after stroke, independent of baseline neurological deficient. Our results may indicate a probable role of corin in stroke prognosis.

## Introduction

Stroke is the leading cause of long-term disability and mortality in China.[1] Better understanding stroke prognostic predictors would undoubtedly improve stroke outcomes. To literature, previous stroke, neurological impairment, diabetes, hypertension, myocardial infarction, and heart failure are potential indicators for stroke mortality, recurrence, and dependency.[2, 3] Although modifications against these risk factors have improved stroke prognosis, some unfavorable outcomes remained unexplained by these well-documented risk factors, [4–6] which has led to a search for new risk factors for stroke prognosis.

Human corin, a type II transmembrane serine protease, is highly expressed by cardiomyocytes.[7, 8] It plays a key role in the regulation of blood volume, blood pressure and cardiac function through activating natriuretic peptides.[9, 10] Corin is shed from the myocyte cell surface, enters the circulation, and is easily measurable.[11] Soluble corin in the circulation has been studied in some disease states, such as heart failure [12], hypertension [13], obesity [14], and pregnant hypertension [15], all of which are associated with stroke prognosis. Recently, we found that decreased serum soluble corin was associated with an increased risk for stroke by a case-control study design.[16] While existing evidence may suggest a probable association between serum soluble corin and stroke prognosis, little is known about the association between serum soluble corin and major disability after stroke in human population. Here, we prospectively followed 522 stroke patients for 3 months to study the prognostic value of serum soluble corin.

## Methods

### Study participants and data collection

A total of 597 acute stroke patients including 481 ischemic and 116 hemorrhagic stroke patients were consecutively recruited after having provided their written informed consent and with approval from the Ethics Committee of Soochow University. At the 3-month follow-up visit, 522 stroke patients were successfully followed (follow-up rate: 87%) and included in the current analysis. The distribution of demographics and risk factors were almost balanced between the 522 followed participants and those loss of follow-up (**S1 Table**). Methods of data collection were described elsewhere [16]. Briefly, this study consecutively recruited patients with first-ever ischemic or hemorrhagic stroke onset within 48 hours confirmed by brain computed tomography or magnetic resonance imaging from 3 hospitals between January 2014 and May 2014. Data on demographic characteristics, lifestyle, medical history, and time from symptom onset to hospitalization were collected at the time of enrollment. Physical examination and blood drawn were also performed at this point. Smoking was defined as having smoked at least 1 cigarette per day for 1 year or more and reported current smoking. Drinking was defined as consuming any type of alcohol beverage at least once per week during the last three years. Three consecutive blood pressure measurements (3 minutes between each) were taken in supine position by trained staff using a standard mercury sphygmomanometer according to a standard protocol [17], after the subjects had been resting for at least 5 minutes. The mean of the three records was used in analysis. Hypertension was defined as blood pressure ≥ 140/90 mmHg and/or use of antihypertensive medication in the last two weeks.[18] Stroke severity was assessed using the National Institutes of Health Stroke Scale (NIHSS) [19] by trained neurologists. Serum soluble corin was measured using a quantikine human corin immunoassay (Catalog: DCRN00; R&D Systems, Inc, Minneapolis). All samples were processed in a duplicate assay. Intra- and interassay coefficients of variation were <2.7% and 6.3%, respectively.

### Follow-up outcomes

A composite outcome consisting major disability at the 3-month follow-up visit, death or vascular events within 3 months after stroke was defined as our study outcome. Major disability was defined as a score of 3 to 5 on the modified Rankin Scale (mRS) at the 3-month follow-up visit.[20] This definition of major disability was also used in many clinical trials. [21] Vascular events included recurrent stroke, myocardial infarction, pulmonary embolism, heart failure, and peripheral arterial disease. Follow-up examination was conducted by neurologists who were blind of baseline characteristics of stroke patients.

### Statistical analysis

Participants were categorized into 3 groups according to tertiles of serum soluble corin distributed in men and women individually because of the sex-difference in serum soluble corin. [12] Baseline characteristics of participants were presented according to these 3 groups. Comparisons among groups were performed using an ANOVA test, Kruskal-Wallis test, or the chi-square test as appropriate. Distribution of study outcomes associated with tertiles of serum soluble corin was examined using the chi-square test. Then stroke patients were categorized into low and high corin groups (the lowest tertile *vs*. the up two tertiles) due to the similar prevalence of study outcomes between the upper two tertiles. To examine the association between serum soluble corin and stroke outcomes, we constructed multivariate logistic regression model in which the composite outcome of stroke was the dependent variable and serum corin level (low *vs*. high) was the independent variable, adjusting for age, sex, baseline NIHSS score, hours from onset to hospitalization, smoking, drinking, hypertension, diabetes, coronary heart disease, atrial fibrillation, family history of stroke, and stroke subtype (hemorrhagic *vs*. thrombotic *vs*. embolic *vs*. lacunar). These variables were adjusted for because they were imbalanced across serum soluble corin tertiles or previously reported to be associated with stroke prognosis. Data analyses were performed using SAS statistical software (version 9.1, Cary, North Carolina). A two-tailed *P* value less than 0.05 was considered statistically significant.

#### Sensitivity analysis

In order to examine whether stroke subtype influenced our results, we conducted separate analysis by stratifying, in addition to adjust for, stroke subtypes (hemorrhagic *vs*. ischemic).

## Results

### Baseline characteristics

As shown in **Table 1**, a total of 522 stroke patients with a mean age of 62 years were studied. Among them, 114 (22%) had a family history of stroke, 51 (9%) suffered from coronary heart disease, and 423 (81%) were ischemic stroke patients. There were 209 (40%) smokers, 135 (26%) drinkers, 330 (63%) hypertensives and 115 (22%) diabetic patients. The median NIHSS score was 4 (interquartile range: 2–8). As expected, body mass index, waist circumference, total cholesterol, and low and high density lipoprotein cholesterol was more common in participants with higher serum soluble corin than those with lower serum soluble corin (all *P* <0.05). Other characteristics were balanced across tertiles of serum soluble corin (all *P* > 0.05).

**Table 1:** Baseline characteristics of stroke patients according to tertiles of serum soluble corin.

Characteristics	Total	Tertiles of serum soluble corin			*P*-value^*^
	(n = 522)	Tertile 1 (n = 166)	Tertile 2 (n = 177)	Tertile 3 (n = 179)	
Age, mean±SD	62.8±12.6	62.8±11.8	62.8±11.8	62.8±11.8	0.975
Male, n (%)	331 (63.41)	105 (63.25)	113 (63.84)	113 (63.13)	0.989
Smoking,%	209 (40.04)	72 (43.37)	75 (42.37)	62 (34.64)	0.188
Drinking,%	135 (25.86)	46 (27.71)	44 (24.86)	45 (25.14)	0.804
Family history of stroke,%	114 (21.84)	36 (21.69)	44 (24.86)	34 (18.99)	0.407
Hypertension, n (%)	330 (63.22)	98 (59.04)	107 (60.45)	125 (69.83)	0.074
Diabetes, n (%)	115 (22.03)	28 (16.87)	35 (19.77)	52 (29.05)	0.016
Coronary heart disease, n (%)	51 (9.77)	19 (11.45)	12 (6.78)	20 (11.17)	0.256
Ischemic stroke, n (%)	423 (81.03)	125 (75.30)	138 (77.97)	160 (89.39)	0.002
Body mass index, mean±SD	24.6±3.7	23.6±3.6	24.4±3.2	25.7±4.2	<0.001
Waist circumference, mean±SD	84.7±11.0	80.9±12.5	85.3±9.5	88.3±9.9	0.001
Systolic blood pressure, mmHg	152.0±23.2	154.2±23.9	151.1±24.5	150.9±21.0	0.336
Diastolic blood pressure, mmHg	87.9±14.5	87.4±13.7	89.0±16.2	87.2±13.2	0.410
Hours from onset to hospitalization	21 (12–33)	20 (12–34)	22 (11–30)	21 (10–35)	0.743
Total cholesterol, mmol/L	4.9 (4.2–5.5)	4.7 (4.0–5.3)	5.0 (4.3–5.8)	5.0 (4.3–5.4)	0.028
Triglycerides, mmol/L	1.4 (1.0–1.9)	1.2 (0.8–1.8)	1.4 (1.0–1.9)	1.4 (1.1–2.0)	0.065
LDL-C, mmol/L	3.0 (2.5–3.6)	2.8 (2.3–3.5)	3.2 (2.5–3.8)	3.1 (2.6–3.5)	0.025
HDL-C, mmol/L	1.1 (0.9–1.3)	1.2 (1.0–1.4)	1.1 (1.0–1.4)	1.1 (0.9–1.2)	0.023
Fasting plasma glucose, mmol/L	6.2 (5.3–7.6)	5.9 (5.3–7.3)	6.3 (5.2–7.8)	6.3 (5.4–8.1)	0.223
Baseline NIHSS score, points	4 (2–8)	4 (2–9)	5 (2–8)	4 (2–7)	0.387

All values are expressed with median (inter-quartile range) unless otherwise noted. HDL-C: high density lipoprotein cholesterol; LDL-C: low density lipoprotein cholesterol; NIHSS: the National Institutes of Health Stroke Scale. Tertile 1: corin <1649 pg/mL for men and corin <1198 pg/mL for women. Tertile 2: 1649 pg/mL ≤ corin <2094 pg/mL for men and 1198 pg/mL ≤ corin <1518 pg/mL for women. Tertile 3: corin ≥2094 pg/mL for men and corin ≥1518 pg/mL for women.

*Comparison across tertiles of serum soluble corin.

### Distribution of prognostic outcomes among tertiles of serum soluble corin

At the 3-month follow-up visit, 158 (30%) patients had unfavorable outcomes of major disability, death, or vascular events. As shown in **Table 2**, we did not find a significant difference in prevalence of any composite outcomes (all *P* >0.05). However, prevalence of the composite outcome of major disability, death, or vascular events (trend *P* = 0.080) and the composite outcome of major disability or death (trend *P* = 0.059) presented a bottom line significant decreasing trend with serum soluble corin tertiles. Interestingly, the prevalence of these outcomes was similar between the second and third teriles of serum soluble corin. This result indicated that stroke patients with such level of serum soluble corin (high corin) had the same prognosis.

**Table 2:** Distribution of prognostic outcomes according to tertiles of serum soluble corin.

Prognostic outcomes		Tertile 1	Tertile 2	Tertile 3	Trend *P*-value
		(n = 166)	(n = 177)	(n = 179)	
major disability, death, vascular events	n (%)	60 (36.14)	49 (27.68)	49 (27.37)	0.080
death or major disability	n (%)	54 (32.53)	42 (23.73)	42 (23.46)	0.059
death or vascular events	n (%)	25 (15.06)	17 (9.60)	21 (11.73)	0.357
death	n (%)	13 (7.83)	8 (4.52)	10 (5.59)	0.390
vascular events	n (%)	17 (10.24)	11 (6.21)	13 (7.26)	0.315

Tertile 1: corin <1649 pg/mL for men and corin <1198 pg/mL for women. Tertile 2: 1649 pg/mL ≤ corin <2094 pg/mL for men and 1198 pg/mL ≤ corin <1518 pg/mL for women. Tertile 3: corin ≥2094 pg/mL for men and corin ≥1518 pg/mL for women.

### Association of high serum soluble corin with prognostic outcomes of stroke

Therefore, we further evaluated the risk for stroke prognosis associated with high corin compared with low corin (the lowest tertile of serum soluble corin). The results were shown in **Table 3**. Patients with high corin had a significantly lower risk for the composite outcome of major disability, death, or vascular events (OR = 0.67, 95%CI: 0.45–0.99) and the composite outcome of major disability or death (OR = 0.64, 95%CI: 0.43–0.96) than patients with low corin. After adjustment for age and baseline NIHSS score, patients with high corin still had a significantly lower risk for the composite outcome of major disability or death (OR = 0.60, 95%CI: 0.36–0.99). After additionally adjusting for other potential confounding factors such as sex, hours from onset to hospitalization, smoking, drinking, hypertension, diabetes, coronary heart disease, atrial fibrillation, family history of stroke, and stroke subtype, odds of the composite outcome of major disability, death, or vascular events (OR = 0.64, *P* = 0.069) and the composite outcome of major disability or death (OR = 0.61, *P* = 0.058) were still lower in high corin group than low corin group, although the *P* values were bottom line significant. We failed to observe a significantly lower risk for other composite outcomes in patients with high corin than those with low corin (all *P* > 0.05).

**Table 3:** Odds ratio and 95% confidence interval for prognostic outcomes according to serum soluble corin level.

Prognostic outcomes	Low corin	High corin	Unadjusted		Age, NIHSS-adjusted		Multivariate-adjusted^*^	
	Cases (%)	Cases (%)	OR (95%CI)	*P*-value	OR (95%CI)	*P*-value	OR (95%CI)	*P*-value
major disability, death, vascular events	60 (36.14)	98 (27.53)	0.67 (0.45–0.99)	0.047	0.65 (0.41–1.03)	0.068	0.64 (0.39–1.04)	0.069
death or major disability	54 (32.53)	84 (23.60)	0.64 (0.43–0.96)	0.032	0.60 (0.36–0.99)	0.047	0.61 (0.36–1.02)	0.058
death or vascular events	25 (15.06)	38 (10.67)	0.67 (0.39–1.16)	0.154	0.70 (0.40–1.23)	0.216	0.67 (0.38–1.21)	0.185
death	13 (7.83)	18 (5.06)	0.63 (0.30–1.31)	0.215	0.66 (0.30–1.46)	0.307	0.62 (0.27–1.41)	0.257
vascular events	17 (10.24)	24 (6.74)	0.63 (0.33–1.21)	0.169	0.65 (0.34–1.25)	0.198	0.62 (0.32–1.22)	0.171

High corin was defined as the upper two tertiles of serum soluble corin. Low corin was defined as the lowest tertile of serum soluble corin.

*Adjusted for age, sex, baseline NIHSS score, hours from onset to hospitalization, smoking, drinking, hypertension, diabetes, coronary heart disease, family history of stroke, atrial fibrillation, and stroke subtype.

### Results of sensitivity analysis

Stratification analysis by ischemic and hemorrhagic stroke showed that the composite outcome of major disability, death, or vascular events (OR = 0.41, *P* = 0.075) and the composite outcome of major disability or death (OR = 0.38, *P* = 0.064) were bottom line significantly associated with serum corin level in hemorrhagic stroke (**S2 Table**). No significant association was found in ischemic stroke. The association between serum soluble corin level and stroke prognosis seemed stronger in hemorrhagic stroke than that in ischemic stroke.

## Discussion

In this study, we followed stroke patients within 3 months after onset to examine the relationship between serum soluble corin and stroke prognosis. We found that serum soluble corin deficiency predicted major disability or death after acute stroke, independent of baseline neurological deficient. Our results suggested a probable role of corin deficiency in stroke prognosis.

This is the first study examining the predictive effect of serum soluble corin on stroke prognosis within 3 months after onset. We previously studied serum soluble corin in stroke patients compared to healthy controls and found a decreased level of serum soluble corin in stroke patients. [16] This result indicated that decreased serum soluble corin was associated with an increased risk for stroke. Our current study showed an association of low corin level with increased risk for poor outcomes of stroke. The current study extended the knowledge of the association between corin and stroke. Corin deficiency may be associated with not only stroke onset but also stroke prognosis.

We previously found that serum soluble corin was lower in hemorrhagic stroke patients compared with ischemic stroke patients. [16] Moreover, as etiologic mechanism is a well-documented predictor of stroke mortality, recurrence and disability [22, 23] and also has important implications for potential treatment differences among patients during the 3 months of follow-up period, this represents an important potential confound for these analysis. As such, we additionally examined the association between serum soluble corin and stroke prognosis among ischemic and hemorrhagic stroke patients separately, adjusting for stroke subtype. The results indicated that serum soluble corin may have a stronger effect on prognosis for hemorrhagic stroke patients than ischemic stroke patients. Considering our previous finding that serum soluble corin is much lower in hemorrhagic stroke than ischemic stroke, serum soluble corin might play a differential role in ischemic and hemorrhagic stroke incidence and prognosis. However, the mechanisms underlying these phenomena are still unclear and merit further study. In addition, we previously found an increased level of serum soluble corin in patients with some cardio-metabolic factors such as hypertension [13], dyslipidemia [24], and obesity [14]. Serum soluble corin was also found to be increased in patients with atrial fibrillation [25] but decreased in patients with heart failure [12] and coronary heart disease [26]. These factors are also established risk factors of stroke risk and prognosis. Serum soluble corin increased in these cardio-metabolic risk factors whereas predicted favorable prognostic outcomes of stroke. This confusing finding indicated a complicated mechanism of corin in stroke risk and prognosis. Detailed studies in respect of molecular mechanisms are needed to clarify the underlined mechanisms. As corin is a primary physiological activator of atrial natriuretic peptide, natriuretic peptides system may be involved in the association between corin and stroke prognosis. So far, accumulating evidence has suggested a prognostic value of atrial natriuretic peptide in predicting post-stroke outcomes.[27, 28]

The study has some limitations that should be discussed. First, the limited sample size resulted in a bottom line significant association between corin level and prognostic outcomes after multivariate adjustment. We did not find any significant associations of continuous serum soluble corin (per SD increment) with stroke outcomes. Therefore, the interpretation of our results should be of cautious. Our results could at least shed light on or generate a hypothesis of the probable role of serum soluble corin in predicting stroke prognosis. Second, patients who were lost of follow-up were not included in current analysis considering the study outcome of major disability which is a constant condition. This may result in underestimation of the association between corin with stroke prognosis, because these patients seem more likely to be disabled and thereby may not come to hospital to receive follow-up examinations. Third, we just measured the concentration of serum soluble corin at one time point after stroke onset. Whether serum soluble corin varies with duration from onset of stroke is unknown, although we did not find significant correlation between serum soluble corin and duration between stroke onset and blood sampling. Serum soluble corin variation after stroke onset may influence our results even though we take hours from onset to blood sampling into consideration in the data analysis. Fourth, we enrolled Chinese patients only. The association of serums soluble corin with stroke prognosis needed to be studied in other populations. Lastly, we did not obtain detailed information on the classification of stroke subtypes. As a result, it is still unclear that which subtype of stroke is more likely to be influenced at a prognostic level by serum soluble corin levels.

In conclusion, serum soluble corin deficiency predicted major disability or death within 3 months after stroke onset. Our results indicated a probable role of serum soluble corin deficiency in stroke prognosis. However, the predicting effect of serum soluble corin on stroke prognosis warranted further study with a larger sample size.

## Supporting Information

S1 Table Baseline characteristics of stroke patients in participants who were followed up and those who were lost of follow-up at the 3-month follow-up after stroke. (PDF)

S2 Table Odds ratio and 95% confidence interval for prognostic outcomes according to serum soluble corin level. (PDF)
