# Supplementary material for: Serum Soluble Corin Deficiency Predicts Major Disability within 3 Months after Acute Stroke
Source: PLoS One. 2016 Sep 22;11(9):e0163731. doi: 10.1371/journal.pone.0163731 (PMC5033232; doi:10.1371/journal.pone.0163731)
Supplement: S1 Table — (PDF) [file pone.0163731.s001.pdf]

S1 Table. Baseline characteristics of stroke patients in participants who were followed up and those who were lost of follow-up at the 3-month follow-up after stroke

Characteristics	Followed up (n=522)	Lost (n=75)	*P*-value*
Age, mean±SD	62.76±12.63	59.25±12.93	0.025
Male, n (%)	331 (63.41)	53 (70.67)	0.220
Smoking,%	209 (40.04)	36 (48.00)	0.190
Drinking,%	135 (25.86)	30 (40.00)	0.011
Family history of stroke,%	114 (21.84)	14 (18.67)	0.531
Hypertension, n (%)	330 (63.22)	47 (62.67)	0.926
Diabetes, n (%)	115 (22.03)	13 (17.33)	0.354
Coronary heart disease, n (%)	51 (9.77)	6 (8.00)	0.626
Ischemic stroke, n (%)	423 (81.03)	58 (77.33)	0.449
Body mass index, mean±SD	24.55±3.73	24.37±2.96	0.706
Waist circumference, mean±SD	84.69±10.99	81.65±8.86	0.156
Systolic blood pressure, mmHg	152.0±23.1	151.2±22.3	0.761
Diastolic blood pressure, mmHg	87.9±14.5	91.9±14.4	0.026
Hours from onset to hospitalization	21 (12-33)	15 (4-34)	0.058
Total cholesterol, mmol/L	4.90 (4.17-5.46)	5.02 (4.36-5.91)	0.192
Triglycerides, mmol/L	1.35 (0.99-1.87)	1.44 (1.12-1.98)	0.492
LDL-C, mmol/L	3.04 (2.51-3.56)	2.94 (2.67-3.88)	0.221
HDL-C, mmol/L	1.12 (0.94-1.34)	1.14 (1.01-1.38)	0.301
Fasting plasma glucose, mmol/L	6.2 (5.3-7.6)	6.1 (5.5-7.3)	0.983
Baseline NIHSS score, points	4 (2-8)	5 (2-7)	0.624
Serum soluble corin, pg/ml	1684 (1305-2077)	1618 (1215-2033)	0.915

All values are expressed with median (inter-quartile range) unless otherwise noted. HDL-C: high density lipoprotein cholesterol; LDL-C: low density lipoprotein cholesterol; NIHSS: the National Institutes of Health Stroke Scale.

*Calculated using student t-test for mean, Kruskal-Wallis test for median, and Chi-square test for percentage.
