# Supplementary material for: Serum Soluble Corin Deficiency Predicts Major Disability within 3 Months after Acute Stroke
Source: PLoS One. 2016 Sep 22;11(9):e0163731. doi: 10.1371/journal.pone.0163731 (PMC5033232; doi:10.1371/journal.pone.0163731)
Supplement: S2 Table — (PDF) [file pone.0163731.s002.pdf]

S2 Table. Odds ratio and 95% confidence interval for prognostic outcomes according to serum soluble corin level

Prognostic outcomes	Low corin	High corin	Unadjusted		Age, NIHSS-adjusted		Multivariate-adjusted*	
	Cases (%)	Cases (%)	OR (95%CI)	*P*-value	OR (95%CI)	*P*-value	OR (95%CI)	*P*-value
Ischemic stroke								
major disability, death, vascular events	41 (32.80)	81 (27.18)	0.76 (0.49-1.20)	0.245	0.86 (0.48-1.55)	0.625	0.88 (0.49-1.61)	0.689
death or major disability	36 (28.80)	68 (22.82)	0.73 (0.46-1.17)	0.193	0.83 (0.43-1.57)	0.559	0.84 (0.43-1.63)	0.605
death or vascular events	18 (14.40)	33 (11.07)	0.74 (0.40-1.37)	0.339	0.81 (0.42-1.56)	0.534	0.87 (0.45-1.68)	0.674
death	8 (6.40)	14 (4.70)	0.72 (0.30-1.76)	0.473	0.80 (0.30-2.13)	0.661	0.91 (0.33-2.54)	0.859
vascular events	13 (10.40)	22 (7.38)	0.69 (0.33-1.41)	0.306	0.73 (0.35-1.51)	0.397	0.76 (0.36-1.61)	0.474
Hemorrhagic stroke								
major disability, death, vascular events	19 (46.34)	17 (29.31)	0.48 (0.21-1.11)	0.085	0.39 (0.15-1.01)	0.052	0.41 (0.15-1.09)	0.075
death or major disability	18 (43.90)	16 (27.59)	0.49 (0.21-1.13)	0.095	0.39 (0.15-1.03)	0.057	0.38 (0.14-1.06)	0.064
death or vascular events	7 (17.07)	5 (8.62)	0.46 (0.14-1.56)	0.212	0.41 (0.11-1.45)	0.165	0.37 (0.09-1.44)	0.151
death	5 (12.20)	4 (6.90)	0.53 (0.13-2.12)	0.372	0.45 (0.11-1.92)	0.282	0.38 (0.07-1.91)	0.239
vascular events	4 (9.67)	2 (3.45)	0.33 (0.06-1.90)	0.214	0.24 (0.04-1.59)	0.138	0.06 (0.00-1.58)	0.093

High corin was defined as the upper two tertiles of serum soluble corin. Low corin was defined as the lowest tertile of serum soluble corin.

*Adjusted for age, sex, baseline NIHSS score, hours from onset to hospitalization, smoking, drinking, hypertension, diabetes, coronary heart disease, family history of stroke, and atrial fibrillation.
